# On the neural networks of empathy: A principal component analysis of an fMRI study

**DOI:** 10.1186/1744-9081-4-41

**Published:** 2008-09-17

**Authors:** Jason S Nomi, Dag Scherfeld, Skara Friederichs, Ralf Schäfer, Matthias Franz, Hans-Jörg Wittsack, Nina P Azari, John Missimer, Rüdiger J Seitz

**Affiliations:** 1 Department of Neurology, University Hospital Düsseldorf, Moorenstrasse 5, 40225, Düsseldorf, Germany; 2 Department of Psychology, University of Hawaii at Hilo, College of Arts and Sciences, 200 W. Kawili Street, Hilo, Hawaii 96720-4091, USA; 3 Clinical Institute of Psychosomatic Medicine and Psychotherapy, University Hospital Düsseldorf, Moorenstrasse 5, 40225 Düsseldorf, Germany; 4 Institute of Diagnostic Radiology, University Hospital Düsseldorf, Moorenstrasse 5, 40225 Düsseldorf, Germany; 5 Paul Scherrer Institute, 5232 Villigen, Switzerland; 6 Biomedical Research Centre, Heinrich-Heine-University Düsseldorf, Moorenstrasse 5, 40225 Düsseldorf, Germany; 7 Brain Imaging Centre West, 52407 Jülich, Germany

## Abstract

**Background:**

Human emotional expressions serve an important communicatory role allowing the rapid transmission of valence information among individuals. We aimed at exploring the neural networks mediating the recognition of and empathy with human facial expressions of emotion.

**Methods:**

A principal component analysis was applied to event-related functional magnetic imaging (fMRI) data of 14 right-handed healthy volunteers (29 +/- 6 years). During scanning, subjects viewed happy, sad and neutral face expressions in the following conditions: emotion recognition, empathizing with emotion, and a control condition of simple object detection. Functionally relevant principal components (PCs) were identified by planned comparisons at an alpha level of p < 0.001.

**Results:**

Four PCs revealed significant differences in variance patterns of the conditions, thereby revealing distinct neural networks: mediating facial identification (PC 1), identification of an expressed emotion (PC 2), attention to an expressed emotion (PC 12), and sense of an emotional state (PC 27).

**Conclusion:**

Our findings further the notion that the appraisal of human facial expressions involves multiple neural circuits that process highly differentiated cognitive aspects of emotion.

## Introduction

Human emotional facial expressions contain information which is essential for social interaction and communication [1-3]. Social interaction and communication depend on correctly recognizing and reacting to rapid fluctuations in the emotional states of others [4,5]. This capability may have played a key role in our ability to survive and evolve [6].

In the research field of psychological childhood development the competence to detect, share, and utilise cognitive patterns and emotional states of the other was conceptualized as mentalizing [7]. The important aspect is that the emotion expressed in someone else's face is not only detected but also valuated from the subjective perspective of the observer. In fact, the appraisal of and the resonance with an emotion observed in somebody else is central to the concept of empathy [8-11].

We can acquire two important sources of information upon perceiving a face: the identification and emotional expression of the individual [12-15]. Support for distinct networks of facial identification and emotional recognition have been provided by studies of those with traumatic brain injuries [16], electroencephalograph (EEG) studies [17,18], magnetoencephalography (MEG) studies [19], and also by studies examining the influence of emotional expression on familiar faces [20]. EEG studies have also hypothesized that these two networks operate in a parallel system of recognition in which emotional effects and structural facial features are simultaneously identified through distinct neural networks [8,9].

When inferring the emotional expression of another, we are automatically compelled to compare our assessment of their emotional state with our own. A key component of perspective taking and emphatic experiences is the ability to compare the emotional state of oneself with another [8-11,21]. However, a distinct separation between first and third-person experiences is necessary [8,22]. People are normally able to correctly attribute emotional states and actions to the proper individual, whether they are our own or someone else's [23]. Confusing our emotional state with another would vitiate the function of empathy and cause unnecessary emotional distress and anxiety [24].

Therefore, a number of distinct psychological states functioning in concert may be necessary in order for a proper emphatic experience to occur [25]. They may include the evaluation of emotion of the self, evaluation of another's emotion, comparing those emotions, and anticipating and reacting to our own or another's emotion, among others. In accord with this line of thought, Decety and Jackson [26] have proposed three major functional components of emphatic experiences. First, the actions of another person automatically enact a psychological state in oneself which mirror those actions and create a representative state which incorporates the perceptions of both the self and another. Secondly, there must be a distinct separation between the perception of the self and the other person. Finally, the ability to cognitively assume the perception of another person while being aware of self/other separation is important.

Each psychological state may be associated with distinct neural networks containing cortical areas which interact within and across neutral networks. The interactions foster an exponentially complex environment within which the processes driving emphatic responses occur. While the processes have been explored to some extent, much work is needed to reveal the precise nature of the complex interaction among the constituents of empathy.

Accordingly, empathy has been suggested to involve distinct, distributed neural networks. Such distributed networks demand the application of a network analysis, which subjects voxels of the image matrix to a multivariate rather than a univariate analysis. This feature allows a network analysis to overcome two significant limitations of univariate analyses that are based on categorical comparisons: they are unable to distinguish regional networks because the constituent voxels may not all change at the defined level of significance and may also show areas of activation which are unrelated to the phenomenon being studied [27].

The network analysis applied to the blood oxygen level dependent (BOLD) signals in this functional magnetic resonance imaging (fMRI) study is a principal component analysis (PCA), which decomposes the image matrix into statistically uncorrelated components. Each component represents a distinct neural network, and the extreme voxel values of a component image, its nodes. Thus, the component images map the functional connectivity of constituent regions activated during neural stimulation. Previous studies have proposed and verified the hypothesis of functional connectivity in regions-of-interest [28] and voxel-based analyses [29-31]. In contrast to regions that show enhanced metabolic or blood flow levels correlated with mental states [31], the networks deduced by PCA incorporate no a priori assumptions regarding the neural stimulation.

Virtually unexplored until now, the neural networks associated with recognizing and empathizing with human facial expressions of emotions are here described using PCA.

We attempt to elucidate the functional neural networks central to the processes of recognizing and empathizing with emotional facial stimuli recorded in fMRI acquisitions of healthy subjects.

Statistical testing identified four principal components (PC) as relevant neural networks. The correlation of the subjects' scores of emotional experience with the PC's further supported their functional relevance. Our analysis shows the coordinated action of brain areas involved in the processing of visual perception and emotional appraisal underlying the facial expressions of human emotions. These regions, including pre-frontal control areas and occipital visual processing areas presumably constitute nodes of the networks implicated in appraising other people's emotion in their facial expressions.

## Methods

### Subjects

Fourteen healthy, right-handed subjects (28.6 +/- 5.5 years; 7 men, 7 women) participated in this study. The subjects had normal or corrected to normal vision. Right-handedness was assessed using the Oldfield's questionnaire [32]. In addition, the subjects' emotional competence was tested with the German, 20-item version of the Toronto Alexithymia Scale (TAS-20) [33-35]. None of the participating subjects were classified as alexithymic (mean TAS-20 sum score 34.14 +/- 6.26, range 23). Subjects were also evaluated with the Beck Depression Inventory [36] and the scales of emotional experience (SEE) [37].

### Visual stimuli

From photographs of facial affect [38], we selected for presentation those with happy, sad and neutral facial expressions found to be correctly identified in more than 90 percent of raters; 14 happy, 14 sad, and 14 neutral faces were used. Only faces which had been found to be correctly identified in more than 90 percent of raters [38] were used to ensure that the subjects internally generated the corresponding emotion. This corresponded to a similar approach of a recent study [39]. As control stimuli we produced a number of scrambled images from these photographs equal to the number of intact faces. All images were digitized and controlled for luminescence.

### Experimental task design

**Figure 1:**
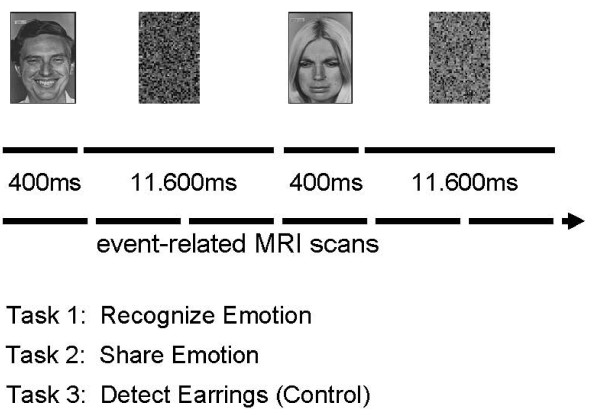
Schematic illustration of the experimental design.

Faces were presented for durations ranging between 300 and 500 ms, since this short presentation time is sufficient for conscious visual perception avoiding habituation [40]. The presentation time of the faces was jittered to enhance the detection of the stimulation-related BOLD activity changes in this event-related fMRI study. Thereafter, scrambled faces were presented for durations between 11 and 12 s (Figure 1). This long second stimulation period was chosen to provide the subjects sufficient time to engage in appraising the emotional facial expression seen in the first stimulation interval and to allow changs of skin conductance to occur [40]. The faces were presented in random order on a laptop connected to a projector (LCD data projector, VPL-S500E, Sony, Toyko, Japan) on a screen which was placed approximately 50 cm from the mirror in the head coil. Immediately before each fMRI scan the subjects were instructed to view the faces in a mind set according to one of the following cognitive instructions: a) identify the emotion expressed in the faces (RECOGNIZE), b) empathize with the emotion expressed in the faces (SHARE EMOTION), c) count the earrings in the faces shown (control condition: DETECT EARRINGS). Each instruction was given twice in random order across the subjects, yielding six separate scans per subject. Thus, the visual stimuli were identical in the different experimental conditions, but the visual information to be processed differed according to the instructions. Since each condition was repeated twice, 28 happy, 28 sad and 28 neutral faces were presented. The presentation was done in random order across the subjects to counteract possible sequence or habituation effects. After each condition, the subjects were debriefed about how well they could perform the task.

### Functional magnetic resonance imaging

The subjects lay supine in the MRI scanner and viewed the faces on a screen via a mirror which was fixed to the head coil. Image presentation was controlled by a TTL-stimulus coming from the MRI-scanner as described in detail elsewhere [41]. Scanning was performed on a Siemens Vision 1.5 T scanner (Erlangen, Germany) using an EPI-GE sequence: TR = 5 s, TE = 66 ms, flip angle = 90°. The whole brain was covered by 30 transaxial slices oriented parallel to the bi-commissural plane with in-plane resolution of 3.125 × 3.125 mm, slice thickness of 4 mm, and interslice gap of 0.4 mm. Each acquisition consisted of 255 volumes. The first 3 volumes of each session did not enter the analysis. A high-resolution 3D T1-weighted image (TR = 40 ms, TE = 5 ms, flip angle = 40°) consisting of 180 sagittal slices with in plane resolution of 1.0 × 1.0 mm was also acquired for each subject.

### Data analysis

Image data were analysed with SPM2 (Wellcome Department of Cognitive Neurology, London, UK; ). Images were slice-time corrected, realigned, normalized to the template created in the Montreal Neurology Institute (MNI), and spatially smoothed with a 10 × 10 × 10 mm Gaussian filter. The normalization step resampled the images to a voxel size of 2 × 2 × 2 mm. The anatomical T1-weighted image of each subject was co-registered to the mean image of the functional images and also normalized to the MNI-space.

For each of the three instruction sets, the happy, sad and neutral face presentations were modelled as well as the corresponding scrambled faces. The models employed the haemodynamic response function provided by SPM2. Data were temporally filtered using a Gaussian low-pass filter of 4 s and a high-pass filter of 100 s. All data were scaled to the grand mean. Realignment parameters as determined in the realignment step were used as confounding covariates. The duration of all events was modelled with 4 s for face presentations and 8 s for the delay period. The repeated condition images of the 18 experimental conditions were averaged for each of the 14 subjects, yielding a total of 252 averaged condition images. Data were modelled using the canonical haemodynamic response function provided by SPM2. The duration of all events was modelled explicitly with 4 s for face presentations and 8 s for the delay period. To identify the brain areas related to viewing the faces a comparison with viewing the scrambled faces was calculated. Only areas with a p < 0.05 corrected at cluster level with a cluster threshold > 20 voxels were accepted (Table 1).

**Table 1:** Cerebral activations related to viewing emotional face expressions as compared with viewing scrambled faces

Anatomical location	Coordinates			Brodmann area	Cognitive instruction (Task)		
	x	y	z		Recognize	Empathize	Object detection

Lingual gyrus R	28	-72	-10	BA 18			+
Fusiform gyrus R	36	-69	-10	BA 19	+	+	
Cuneus R	28	-78	16	BA 31	+	+	
Superior frontal gyrus L	-3	22	52	BA 6	+	+	+
Inferior frontal gyrus L	-49	2	20	BA 44	+	+	+
Inferior frontal gyrus R	55	8	16	BA 44	+	+	+
Middle frontal gyrus R	43	29	30	BA 9	+	+	
Supramarginal gyrus L	-46	-49	28	BA 42	+		
Parietal operculum R	50	-24	10	BA 40	+		
Sup. temporal gyrus R	34	16	-6	BA 38	+		
Hypothalamus L	-3	-6	-11		+		
Cerebellum L	-14	-60	-22				+

Activations in categorical analysis with SPM2 at p < 0.05 FDR with clusters > 20 voxels. The coordinates (x, y, z; mm) of the peak activations (+) are listed in stereotactic space [43]. L refers to left hemisphere; R refers to right hemisphere.

The PCA employed in house software of which some modules were adapted from SPM2. Extracerebral voxels were excluded from the analysis using a mask derived from the gray matter component yielded by segmentation of the high resolution anatomical 3D image volume into gray matter, white matter and cerebrospinal fluid using the segmentation module of SPM2. Voxel values of the segmented image ranged between 0 and 1; the mask included only those exceeding 0.35, excluding most of white matter. Calculation of the residual matrix is the first step. From a matrix whose rows corresponded to the 252 conditons and columns to the 180 thousands voxels in a single image volume the mean voxel value of each row was subtracted from each element as was the mean voxel value of each column subtracted from each element. Thereafter, the grand mean of all voxel values in the original matrix was added to each element. The result of this normalization procedure is the residual matrix for which the row, column and grand means vanish. Using the singular value decomposition implemented in Matlab, the residual matrix was then decomposed into 252 components, consisting of an image, an expression coefficient, and an eigenvalue for each component. The eigenvalue was proportional to the square root of the fraction of variance described by each component, while the expression coefficients described the amount that each subject and condition contributed to the component. The principal components (PCs) were ranked according to the proportion of variance that each component explains, i.e, PC 1 explains the greatest amount of variance. The expression coefficients and voxel values of a PC were orthonormal and their orthogonality reflected the statistical independence of the PCs. The PC image displays the degree to which the voxels covaried in each PC, their voxel values (loadings) ranged between -1 and 1.

In order to provide a neurophysiological interpretation of the components, statistical tests, e.g. unpaired t-tests and tests of correlation (Pearson) were applied to the expression coefficients. The formal criteria for relevant PCs were: (1) the statistical tests identified the condition differentiating PCs at a significance level of p < 0.001, and (2) the PCs fulfilled the Guttman-Kaiser criterion, the most common retention criterion in PCA [42] in which PCs associated with eigenvalues of the covariance matrix larger in magnitude than the average of all eigenvalues are retained, implying in this analysis that they ranked among the first 61 PCs. The voxels describing the nodes of a neural network associated with a relevant PC image volume fulfilled the conditions that the voxel values lie in the 1^st ^percentile or the 99^th ^percentile of the volume's voxel value distribution, and that the voxels belong to clusters of greater than 50 voxels.

The anatomical locations of the peak activations and of the coordinates of the maximal PC loadings of the significant PCs are reported in Talairach space [43]. A freely distributed Matlab script [44] effected the transformation from MNI space.

Correlation of the expression coefficients of the significant PCs with the TAS-20, the Beck Depression Inventory, and the SEE scales was conducted using a Pearson two-tailed correlation (SPSS for Windows, Version 12.0.1.). The significance level of the correlations was set to p < 0.01.

## Results

Before the fMRI experiment the subjects' capacity to experience emotions was tested with the German, 20-item version of the Toronto Alexithymia Scale (TAS-20 [35]). It was found that the 14 participating subjects had a normal mean TAS-20 sum score (34.1 +/- 6.3, range 23). This indicated that each of the subjects had a high capacity of introspection and emotional awareness. In the fMRI session the subjects stated that they could readily identify the seen emotion and generate the corresponding emotion internally as instructed. They detected 92 +/- 0.2 percent of the faces wearing earrings.

The categorical analysis showed that viewing the emotional face expressions as compared with viewing the scrambled faces resulted in activations of right visual cortical areas and bilaterally the inferior frontal and superior frontal gyrus (Table 1). Recognizing emotional facial expressions showed the most extensive activation pattern involving also the hypothalamus, the left supramarginal gyrus, cortical areas at the right temporal parietal junction, and the left hypothalamus (Table 1). Empathizing with the seen emotion as compared with object detection (control condition) resulted in one activation area which occurred in the left inferior frontal gyrus. Note, that no activation occurred in the anterior prefrontal or orbitofrontal cortex.

**Figure 2:**
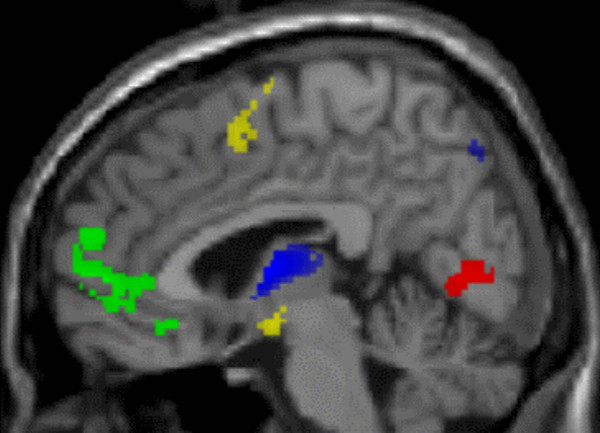
Brain areas involved in the PC1 and PC2 superimposed on the canonical single-subject MR image of SPM2 in a sagittal plane showing the areas involved in PC1 (cuneus: red – negative loading; anterior portion of superior frontal gyrus: green – positive loading) and in PC2 (precuneus, thalamus: blue – negative loading; pre-SMA, hypothalamus: yellow – positive expression loading).

**Figure 3:**
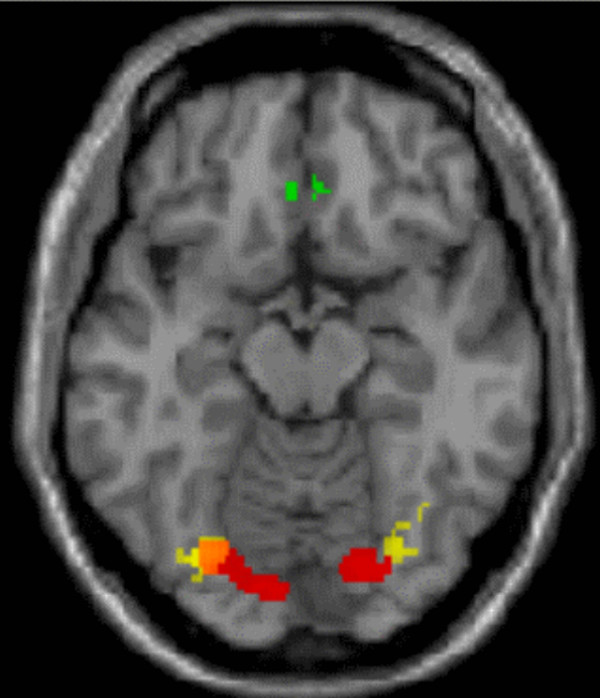
**Brain areas involved in PC1 and PC2 superimposed on the canonical single-subject MR image of SPM2 in an axial plane showing the lateral position of activity in the fusiform face area (PC2, yellow, positive loading) relative to PC1 (red, negative loading).** Note the medial prefrontal involvement in PC1 (green, positive loading).

The network analysis revealed that out of the total of 61 retained PCs four differentiated the experimental conditions as found by formal statistical testing (Table 2); for the 11 statistical tests described below, the probability threshold corrected for multiple comparisons is p < 0.001. PC1 explained 16.8 percent of the variance and distinguished between viewing the happy, sad and neutral faces from viewing the scrambled faces during RECOGNIZE, SHARE EMOTION and DETECT EARRINGS. Accordingly, PC1 represented a neural network associated with face identification. The areas with the positive loadings included the right dorsolateral and superior frontal cortex, the left anterior cingulate, and bilaterally the inferior parietal region. The areas with the negative loading included bilaterally the lingual gyrus, the precuneus, and the cuneus, which are areas involved in higher order processing of visual information (Table 2, Figures 2 and 3).

**Table 2:** Cerebral circuits in processing of emotional face expressions

	Coordinates			Anatomical location	Broadmann area	Cluster size
	X	Y	Z			

PC1: *Face identification *(16.8%)						
Positive loadings	4	53	4	Superior frontal gyrus R	BA 10	248
	-4	48	-2	Anterior cingulate L	BA 32	81
	1	31	-7	Anterior cingulate R	BA 32	76
	38	26	45	Middle frontal gyrus L	BA 8	66
	-50	-63	31	Angular gyrus L	BA 39	53
	44	-61	28	Supramarginal gyrus R	BA 39	396
Negative loadings	-21	-77	-10	Lingual gyrus L	BA 18	306
	28	-72	-10	Lingual gyrus R	BA 18	314
	34	-70	15	Cuneus R	BA 31	428
	-6	-75	44	Precuneus L	BA 7	54
						
PC2: *Identification of expressed emotion *(4.7%)						
Positive loadings	-30	-74	-10	Fusiform gyrus L	BA 19	158
	36	-69	-13	Fusiform gyrus R	BA 19	252
	40	-87	8	Middle occipital gyrus R	BA 19	60
	6	3	59	Superior frontal gyrus L	BA 6	87
	-49	2	20	Inferior frontal gyrus L	BA 44	65
	-3	-6	-11	Hypothalumus L		50
	4	-6	-11	Hypothalumus R		63
Negative loadings	-5	-73	46	Precuneus L	BA 7	51
	2	-63	52	Precuneus R	BA 7	71
	-63	-10	0	Superior temporal gyrus L	BA 22	66
	-2	4	6	Thalamus L		259
	7	-3	6	Thalamus		300
	-14	-30	-22	Pons L		80
	50	-60	-27	Neocerebellum R		71
						
PC 12: *Attention to expressed emotion *(1.6%)						
Positive loadings	4	-86	17	Cuneus R	BA 18	230
	54	8	36	Middle frontal gyrus R	BA 9	115
	55	17	16	Inferior frontal gyrus R	BA 44	56
	-51	-35	-2	Middle temporal gyrus L	BA 22	64
	59	-56	3	Middle temporal gyrus R	BA 22	119
	8	-8	12	Thalamus R		377
Negative loadings	-12	-77	48	Precuneus L	BA 7	117
	8	-69	48	Precuneus R	BA 7	68
	-42	-48	56	Inferior parietal lobule L	BA 40	128
	-32	60	6	Middle frontal gyrus L	BA 10	246
	43	29	30	Middle frontal gyrus R	BA 9	132
						
PC 27: *Sense of emotional state *(0.9%)						
Positive loadings	-48	-57	-6	Middle occipital gyrus L	BA 37	68
	-28	52	21	Middle frontal gyrus L	BA 10	153
	-5	5	0	Caudate L		66
	-13	-18	-25	Pons L		131
	-1	-47	-36	Medulla oblongata		192
Negative loadings	-42	-59	-17	Fusiform gyrus L	BA 37	58
	-46	-49	28	Supramarginal gyrus L	BA 42	86
	51	-34	50	Inferior parietal lobule R	BA 40	54
	55	-19	16	Parietal operculum R	BA 40	53
	-55	-40	8	Superior temporal gyrus L	BA 22	162
	38	8	-26	Superior temporal gyrus R	BA 38	59

Shown are the relevant principal components (PC) and their designations. Indicated in the parentheses beside each PC entry is the amount of variance accounted for by that PC. The coordinates (x, y, z; mm) are listed in stereotactic space [43]. Only those voxels with values lying in the 99^th ^percentile (positive loadings) or the 1^st ^percentile (negative loadings) and in cluster with sizes greater than 50 voxels were included. L refers to left hemisphere; R refers to right hemisphere.

PC2 explained 4.7 percent of the variance and differentiated viewing the happy, sad, and neutral faces from viewing scrambled faces during the RECOGNIZE and SHARE EMOTION conditions (Table 2, Figures 2 and 3). Therefore, PC2 was expected to depict a neural network associated with the identification of an expressed emotion. In fact, the areas with positive loadings included bilaterally the fusiform gyrus, the right middle occipital gyrus, the right superior frontal gyrus, and the left inferior frontal gyrus. The areas with negative loadings included bilaterally the precuneus, the left superior temporal gyrus, as well as the thalamus, pons, and neocerebellum (Table 2). The relative localization of the cortical areas in the occipital cortex and the superior frontal gyrus involved in PC1 and PC2 is illustrated in Figure 3.

PC12 explained 1.6 percent of the variance and reflected the contrast of the conditions SHARE EMOTION and DETECT EARRINGS as well as the contrast of the conditions RECOGNIZE and DETECT EARRINGS during and after viewing happy, sad, or neutral faces. The comparisons showed that PC12 represented a neural network associated with the subjects' attention to an expressed emotion. Areas involved in this network included the right cuneus, bilaterally the precuneus, middle frontal and temporal gyrus, and left inferior parietal lobule (Table 2).

**Figure 4:**
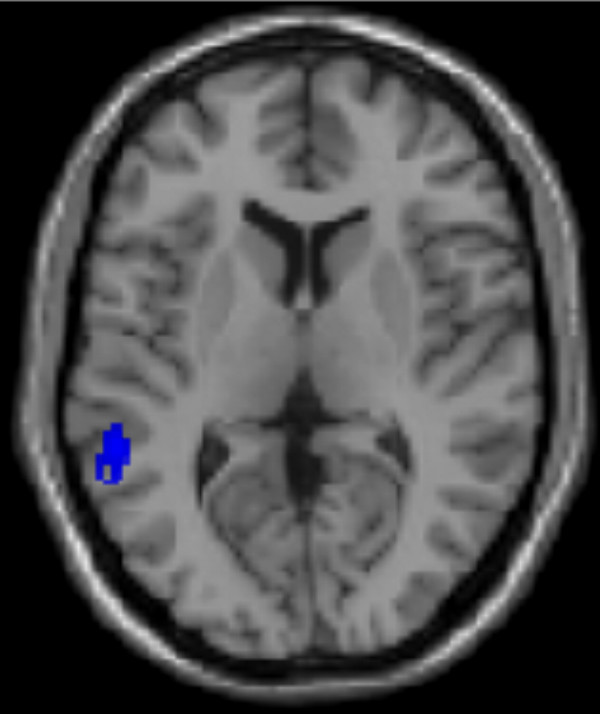
Involvement of the left superior temporal gyrus in PC27 superimposed on the canonical single-subject MR image of SPM2 in an axial plane (blue, negative loading).

PC27 explained 0.9 percent of the variance and represented the contrast of the conditions SHARE EMOTION with RECOGNIZE during and after viewing happy, sad, and neutral faces. We hypothesized, therefore, that this PC characterizes the neural network subserving the sensation of the emotional state associated with empathy. The regions involved were the left fusiform and middle occipital gyrus, the left middle frontal gyrus, bilaterally the inferior parietal lobule and the superior temporal gyrus (Table 2, Figure 4). Subcortical structures, such as the left caudate and brain stem were also involved.

**Figure 5:**
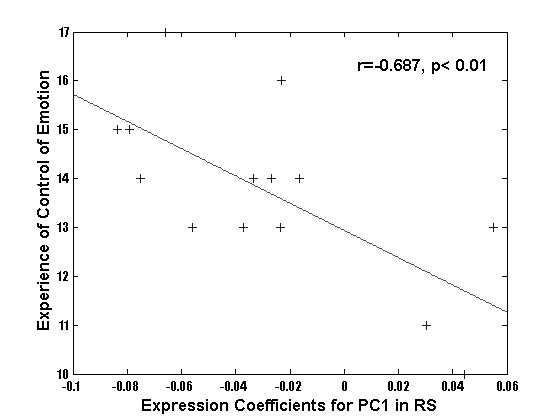
Regression plots of expression coefficients of PC1 with data of the test scores of the 14 subjects highlighting the functional relevance of these PCs.

**Figure 6:**
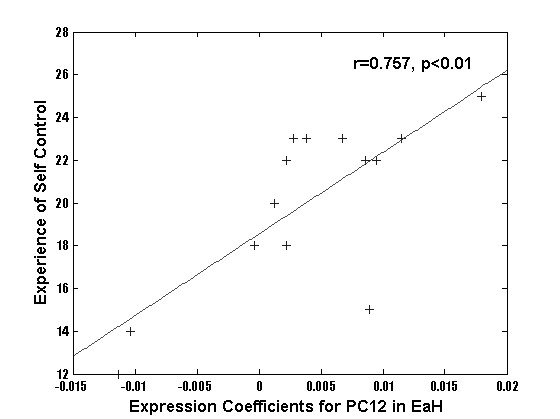
Regression plots of expression coefficients of PC12 with data of the test scores of the 14 subjects highlighting the functional relevance of these PCs.

Correlation of the PC expression coefficients with the behavioral scales of emotional processing yielded the following observations (Table 3). Beck's depression inventory subject scores correlated significantly with the PC2 expression coefficients computed for the RECOGNIZE condition after viewing neutral faces. Since none of the subjects exhibited score values suggestive of depression, this correlation was obtained in the normal range of the Beck's depression inventory. Nevertheless, a more negative emotional experience was related to recognizing neutral faces. Further, the TAS-20 scores correlated negatively with PC2 expression coefficients computed for viewing happy and neutral faces in the control condition DETECT EARRINGS. Note, that the TAS-20 classified all subjects as highly emotionally sensitive. Thus, this correlation suggests that the more sensitive to processing of emotion our subjects were, the more they were so during implicit processing of faces. The SEE-scale score values related to experience of emotional control correlated negatively with PC 1 expression coefficients computed for viewing of sad faces in the RECOGNIZE condition (Figure 5). A similar correlation was found for PC27 expression coefficients computed for viewing of neutral faces in the RECOGNIZE condition. This suggested that recognizing sad and neutral faces was most pronounced in the subjects whose scores indicated relatively impaired emotional control. Finally, the SEE-scale score values of experience of self-control correlated with PC 12 expression coefficients computed for the SHARE EMOTION condition after viewing happy and neutral faces (Figure 6). This suggested that subjects with a high level of self-control most strongly empathized with happy and neutral faces. Thus, viewing sad faces may have impaired the subject's perception of emotional control, while processing the happy face expression appears to have improved it (Table 3). In contrast, the processing of neutral faces was related to a relatively negative emotion, possibly due to the ambiguous character of the neutral faces (Table 3).

**Table 3:** Correlation of behavioral data vs. expression coefficients

*Toronto alexthymia scale-20*		*Beck depression inventory*		*Experience of control of emotion*		*Experience of self control*	
R value	P value	R value	P value	R value	P value	R value	P value
							
Condition/PC		Condition/PC		Condition/PC		Condition/PC	
							
*CH 2 *-0.670	0.009	*RaN 2 *0.667	0.009	*RS 1 *-0.687	0.007	*EaH 12 *0.757	0.002
							
*CN 2 *-0.683	0.007			*RN 27 *-0.675	0.008	*EaN 12 *0.778	0.001

C = DETECT EARRINGS (Control), E = SHARE EMOTION, R = RECOGNIZE, H = Happy, S = Sad, N = Neutral, aH = after Happy, aN = after Neutral

## Discussion

The novel finding of this event-related fMRI study is that recognizing and empathizing with emotional face expressions engaged a widespread cortical network involving visual areas in the temporal and occipital cortex which are known to be involved in face processing [12,45-62]. Previous fMRI studies have identified several brain regions that are consistently activated when perceiving facial stimuli. These regions include the fusiform gyrus, which is often referred to as the "fusiform face area" [45,49-55,61-63], a face-selective region in the occipito-temporal cortex [45-48,56-60], and the superior temporal sulcus [12,13,48,52].

Because the involved cortical areas are active within and across multiple neural networks, the difficulty of assigning a single function to a cortical area becomes apparent [2]. In fact, disagreement over assigning functions to cortical areas does exist, with some arguing that function may be tied to elements of experiment design [64]. However, inability to agree over the specific function of cortical areas would further suggest that a description of "face network" or "face pathway" may be more accurate than "face region" [65].

Notably, cortical areas in the anterior lateral and medial prefrontal cortex known to participate in manipulating and monitoring information and controlling behavior were also involved [26,66]. Multivariate image analysis using PCA permits the characterization of different networks that include brain areas changing brain activity in related to a given task and brain areas contributing to the function without necessarily changing their activity [28-31,67]. Specifically, we applied inferential statistical tests to identify the PCs that effectively differentiated between the experimental conditions [31,68]. The four thereby identified PCs showed more cerebral areas involved than in the simple task comparions. These PCs revealed correlations with behavioral data obtained prior to the fMRI acquisitions, which highlighted their functional relevance.

### Functional neural networks

Of the four differentiating PCs, PC1 distingished the neural network involved in face indentification, since the involved lingual gyrus, cuneus and precuneus have been known to be involved in face recognition and visual processing [68,69]. The lingual gyrus has been linked to an early stage of facial processing which occurs before specific identification occurs [70]. Conversely, the angular gyrus and the anterior cingulate have been shown previously to be involved in attention [71], and the anterior portion of the superior frontal gyrus has been implicated in theory of mind paradigms [26,71].

PC2 represented the functional neural network associated with detecting emotional facial expressions, since the network nodes included bilaterally the fusiform gyrus, corresponding to the so-called fusiform face area. Some studies have suggested that the fusiform face area mediates the lower order processing of simple face recognition [72], while others have implicated it in higher order processing of faces at a specific level [45] including emotional detection [73] and identity discrimination [58,64]. Also included among the nodes were the posterior portion of the superior frontal and the inferior frontal gyrus, which have been implicated in higher order processing of faces involving the perception of gaze direction [74], attention to faces [75], eye and mouth movements [56], and empathy [25,76]. Thus, our analysis, relating the concerted action of the fusiform and superior frontal gyrus to the detection of emotional face expressions, substantiates previous work showing that the conjoint activity of the fusiform gyrus and cortex lining the superior temporal sulcus are related to higher order processing of facial features [45,53,56,58,64,72-74]. We propose that these areas constitute a system involved in facial affect processing.

PC2 also implicated subcortical structures such as the thalamus and the hypothalamus bilaterally, and the cerebellum. These structures belong to the anterior cingulate – ventral striatum – thalamus – hypothalamus loop [76-78] and are thought to regulate the emotions, drives, and motivated behavior [2,79,80].

The third statistically relevant PC, PC12, was related to the attentive processing of expressed emotions. Accordingly, the involved cortical areas, e.g. the precuneus, cuneus, middle frontal gyrus, and thalamus have all been linked to tasks related to controlling attention [81-83]. The inferior frontal gyrus has been shown to be involved in response inhibition during attention [84], while the middle temporal gyrus has been implicated in attention to facial stimuli [47] and to visual attention in general [81,82].

PC27 appeared to represent a network mediating a 'feeling' or sense of an observed emotional state. In fact, the superior temporal gyrus has been shown to play an important role in perceiving self/other distinctions, and, importantly, in experiencing a sense of agency [85,86]. Also, the superior temporal gyrus seems to be important for "theory of mind" capabilities [87-89]. Similarly, the right inferior parietal lobule has been related to an experienced sense of agency [85,86], while the temporal parietal junction has been thought to be crucial to larger networks mediating spatial unity of the self and body [89,90], and attention [81,91,92].

### Activity within and across functional networks

Empathic processes allow individuals to quickly asses the emotional states and needs of other individuals while rapidly transmitting our own experiences and needs [93]. Thus, emphatic experiences are crucial to rapid and successful social interaction [94,95]. The recognition of emotional states and cognitive processing of another's emotional expression are critical skillsets utilized by all humans upon perceiving another person's face. In order to properly behave in a social situation, one must understand the *context *of the situation before an appropriate action can be taken.

The participation of the prefrontal cortex in the four relevant PCs in our study not only support the view that different cognitive functions work together to play a role in distinct neural networks, but, as well, further the notion that the medial pre-frontal cortex is the junction point where different visual, attentive, emotional and higher order cognitive processes come together in order to allow for a subjective reaction towards the exterior world [26]. The medial-pre-frontal integration of own cognitive concepts, implicit affective schemata and empathy-based anticipation of the possible reactions of important other individuals allows for an effective selection and adaptive planning of one's own actions. The face as the most significant carrier of emotional information is an important source for these evaluative and appraisal functions.

The four neural networks involved in the recognition of faces and the cognitive control of emotions capture the functions that this study aimed to explore. Our results support numerous studies that hypothesize distinct neural networks for recognizing facial features and emotional expressions [11,13-17,96,97]. In particular, although PC 1 and PC 2 differentiated lower-order facial identification and higher-order facial feature processing, the cortical areas within each PC contained brain areas associated with functions effected by the neural networks depicted in the other PCs. Thus, the middle frontal gyrus activated in all four principal components has been shown to be related to inhibition [98], successful recognition of previously studied items [99], and successful error detection, response inhibition, interference resolution, and behavioral conflict resolution associated with completing the Stroop Color-Word task [100]. Moreover, the PC representing a basic visual function, PC1 included activation of the right anterior cingulate which has been shown to severely impact the processing of emotional expressions [101,102]. Apparent also in PC1, the lingual gyrus and cuneus have been implicated in the higher-order processing of emotional expression [103] and the lingual gyrus has been implicated in motional information [104]. Interestingly the anterior cingulate has also been thought to cognitively monitor the control of response conflict in information processing [105] and in the regulation of cognitive and emotional processing [106]. In a case study, Steeves et al. [61] demonstrated that an intact fusiform face area was sufficient for identifying faces, but a lesion in the occipital face area prevented the patient from higher-order processing of faces such as identity, gender, or emotion.

Out results are also in accordance with the idea that emotion recognition may play a role in the lower order process of face identification, but this role is limited at best and functions mainly as a stepping stone for higher order recognition of emotional expressions. [107]. Although the lingual gyrus in PC1 and the fusiform face area in PC2 are located closely anatomically, our analysis suggests that they are involved in distinct neural networks related to different subfunctions, face identification and detection of emotional facial expressions, repectively, of facial processing. Nevertheless, it is possible that the subjects recognized the emotions implicitly also in the DETECT EARRING condition. However, the cognitive instruction in this study was that during the RECOGNIZE and SHARE EMOTION conditions the subjects had to appraise the emotions explicitly. In fact, this difference resulted in additional brain structures involved in the explicit processing conditions.

The involvement of closely adjacent cortical areas in different subfunctions related to the detection of emotional face expressions highlights the difficulty of assigning exclusive functional relevance to a particular area and suggests the multi-functional nature of brain areas which participate in multiple, interconnected neural networks performing lower-order as well as higher-order neural processing.

## Conclusion

Implemented in this study using PCA, employment of a network analysis helps to elucidate the coordination of multiple cortical areas in brain functions. We are able to identify the participation of multiple neural networks in processing highly differentiated cognitive aspects of emotion. Ultimately, placing cortical areas within the *context *of a particular neural network may be the key to defining the functional relevance of individual cortical areas. This more sensitive approach gives us a better picture of the constituent components involved in the processes of recognizing and empathizing with emotional facial expressions by discerning networks of activity, rather than simply defining areas of activity [28,29]. This in turn gives us a better idea of how the functional connectivity of constituent regions work together in order to allow a person to recognize and process the facial expressions of another.

## Competing interests

The authors declare that they have no competing interests.

## Authors' contributions

JSN performed the statistical analysis and drafted the manuscript. DS participated in the design of the study, programmed the stimuli and performed the acquisition of the fMRI data. SF participated in the fMRI experiment and the statistical analysis. RS participated in the design of the study and performed the psychological investigations. MF participated in the design of the study and contributed to the interpretation of the data and drafting of the manuscript. HJW participated in the design of the study and participated in the acquisition of the fMRI data. NPA contributed to the design of the study and participated in the interpretation of the data and drafting of the manuscript. JM performed the statistical analysis and participated in the interpretation of the data. RJS participated in the design of the study, contributed to the statistical analysis, the data interpretation, and drafting the manuscript. All authors read and approved the final manuscript.
